# Agenesis of Dorsal Pancreas in a Young Adult: A Case Report

**DOI:** 10.31729/jnma.8304

**Published:** 2023-10-31

**Authors:** Nistha Ulak, Manjil Bharati, Suju Bhattarai, Philip Shyam Ranjit, Rabina Byanju

**Affiliations:** 1 Department of Internal Medicine, Kathmandu University School of Medical Sciences, Dhulikhel, Kavrepalanchok, Nepal; 2 Department of Internal Medicine, Kathmandu Medical College and Teaching Hospital, Sinamangal, Kathmandu, Nepal; 3 Department of Internal Medicine, B&B Hospital Pvt. Ltd, Gwarko, Lalitpur, Nepal

**Keywords:** *agenesis*, *case reports*, *congenital*, *pancreas*

## Abstract

Dorsal agenesis of the pancreas is an exceedingly rare congenital anomaly where the dorsal part of the pancreas fails to develop properly during embryonic development. We report a case of partial agenesis of the dorsal pancreas in a 27-year-old female who presented with progressively worsening abdominal pain, nausea, vomiting, generalized weakness, easy fatigability, and dizziness. Physical examination revealed fair general condition with stable vital signs and normal abdominal and other system findings. Laboratory evaluations and abdominal computed tomography scan revealed an absence of the pancreatic body, tail, and duct of Santorini, confirming the diagnosis of partial agenesis of the dorsal pancreas. Only around 100 cases have been reported in the literature, making it a diagnostic challenge. Our case illustrates the rarity and challenges in diagnosing dorsal agenesis of the pancreas. Further research is needed to fully understand its causes and associations.

## INTRODUCTION

Agenesis of the dorsal pancreas (ADP) is a rare congenital anomaly in which the dorsal part of the pancreas fails to develop properly during fetal development.^1^ The ventral and dorsal buds generate the pancreas during development and eventually unite to form a single organ. The dorsal bud either never forms or totally degenerates in cases of dorsal pancreas agenesis.^2^ Fewer than 100 cases have been reported in the literature so far.^1^ ADP may be discovered when evaluating conditions arising from anomalies like pancreatitis and diabetes mellitus. Here, we report a case of a 27-year-old female with partial agenesis of the dorsal pancreas.

## CASE REPORT

A 27-year-old female presented to the Outpatient Department with a 5 day history of progressively worsening abdominal pain. The pain was mainly localised over the epigastrium and left flank and was relieved only after receiving intravenous medications. She also experienced multiple episodes of vomiting that contained food particles and were not bile-or blood-stained. The patient reported a decrease in appetite, generalised weakness, easy fatigability, and

dizziness. Additionally, she complained of retrosternal discomfort and a foreign body sensation in the upper one third region of the chest. The patient had a history of altered bowel habits, with alternating constipation and diarrhoea. She had been admitted to the hospital twice before for the same complaints, and each time, the symptoms were managed conservatively. Upon examination, the patient appeared to be in fair general condition with stable vital signs. Her heart rate was 84 beats per minute, her respiratory rate was 22 breaths per minute, her blood pressure was 120/80 mmHg, and her oxygen saturation was 99% in room air. An abdominal examination revealed a soft, non-tender abdomen with normal bowel sounds. Other system examinations, including chest and cardiovascular findings, were unremarkable.

**Figure 1:**
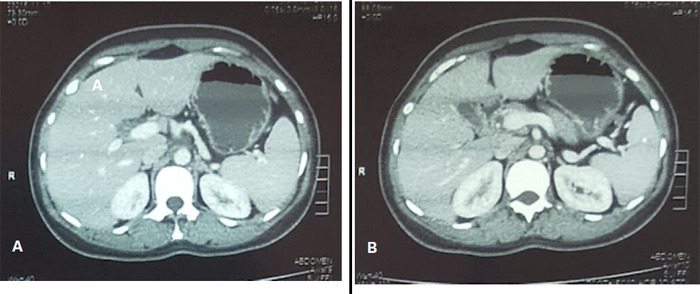
Abdominal CT scan reveals an absence of the body and tail of the pancreas.

Laboratory evaluation revealed a white blood cell count of 6,810/mm^3^, haemoglobin 12.46 g/dL, platelet count of 261,500/mm^3^, serum albumin 3.5 g/dL, aspartate aminotransferase 30 U/L, alanine aminotransferase 26 U/L, and alkaline phosphatase 67 U/L. The total bilirubin was 0.4 mg/dL with a 0.2 mg/dL direct fraction. Serum amylase and lipase were within the normal range. An abdominal computed tomography (CT) scan showed a normal-appearing pancreatic head and the complete absence of the neck, body, and tail (Figure 1).

Upper gastrointestinal endoscopy revealed reflux esophagitis with erosive gastritis. The patient was treated with intravenous antibiotics, proton pump inhibitors, anti-emetics, and other supportive therapies. Additionally, a surgical consultation was obtained. After symptomatic treatment, she was discharged. During follow-up, the patient feels better with the above-mentioned treatment and is later asymptomatic without medication.

## DISCUSSION

Embryologically, the pancreas is derived from dorsal and ventral endodermal buds. The ventral bud forms the head (posteroinferior part) and uncinate process and gives rise to the Wirsung duct/main duct which drains through the major papilla. The dorsal pancreatic bud forms the body and the tail of the pancreas and gives rise to the accessory pancreatic duct (Duct of Santorini) which drains through the minor papilla.^1^ Any failure in the development of the dorsal bud thus leads to an absence of a functional pancreatic body, tail, and duct of Santorini. This anomaly could be partial or complete. In partial ADP, the minor papilla, duct of Santorini, or the pancreatic body are present. In complete ADP, the neck, the body, the tail of the pancreas, the duct of Santorini, and the minor papilla are all absent.^3^

ADP is mostly asymptomatic, but common presenting symptoms include diabetes mellitus, abdominal pain, pancreatitis, enlarged pancreatic head, and, in a few cases, polysplenia.^4^ In a few instances, dorsal pancreatic agenesis remained a diagnostic challenge in the evaluation of abdominal pain.^5^ The exact genetic pathogenesis of ADP is still unknown. Some literature suggests that Hepatocyte Nuclear Factor 1-beta and GATA Binding Protein 6 genes were proven to be correlated with the embryogenic development of the pancreas.^6^ However, experiments in mice showed that mutation in retinaldehyde dehydrogenase 2 (Raldh2) and gene H1xb9 or deficiency of retinoic acid resulted in ADP.^7^

A few cases of dorsal pancreatic agenesis that presented with chronic pancreatitis are also reported.^8^ However, our patient did not report any symptoms of chronic pancreatitis. In a case of dorsal pancreatic agenesis, in a woman with diabetes mellitus and both of her sons, the pattern of genetic transmission for this anomaly was either autosomal dominant or x-linked dominant.^9^ However, in our case, there was no associated family history with the patient. As indicated in the literature, dorsal pancreatic agenesis has been linked to various organ anomalies such as multiple splenic malformations, Kartegener syndrome, polycystic kidney disease, congenital choledochal cysts, and biliary atresia.^10^ Furthermore, neither of the other anomalies was found in our case. In our case, the finding of dorsal pancreatic agenesis is incidental. The patient does not have any other findings or associated comorbidities, as mentioned in the literature. The rigid association has not yet been proved, despite the fact that a small number of research link genetic pathways to its development.

Thus, as noted in the earlier literature, our case demonstrates that the dorsal pancreatic agenesis may be an incidental finding with no accompanying characteristics or an odd presentation and further research on its causation and connection is required to fully establish the pathogenesis.
